# Variations in amount of TSST-1 produced by clinical methicillin resistant *Staphylococcus aureus *(MRSA) isolates and allelic variation in accessory gene regulator (*agr*) locus

**DOI:** 10.1186/1471-2180-9-52

**Published:** 2009-03-10

**Authors:** Miki Nagao, Akira Okamoto, Keiko Yamada, Tadao Hasegawa, Yoshinori Hasegawa, Michio Ohta

**Affiliations:** 1 Department of Infection Control and Prevention, Kyoto University Hospital, Kyoto, Japan; 2 Department of Molecular Bacteriology, Nagoya City University Graduate School of Medical Science, Nagoya, Japan; 3 Department of Respiratory Medicine, Nagoya University Graduate School of Medicine, Nagoya, Japan

## Abstract

**Background:**

*Staphylococcus aureus *(S. aureus) is an important pathogen associated with both nosocomial and community-acquired infections and its pathogenicity is attributed to its potential to produce virulence factors. Since the amount of toxin produced is related to virulence, evaluating toxin production should be useful for controlling S. aureus infection. We previously found that some strains produce relatively large amounts of TSST-1; however, no reports have described the amount of TSST-1 produced by clinical isolates.

**Methods:**

Amounts of TSST-1 produced by clinical methicillin resistant S. aureus (MRSA) isolates were measured by Western blotting. We determined their accessory gene regulator (*agr*) class by PCR and investigated whether TSST-1 production correlates with variations in the class and structure of the *agr*.

**Results:**

We found that 75% of surveyed MRSA isolates (n = 152) possessed the *tst *gene and that 96.7% belonged to *agr *class 2. The concentrations of TSST-1 secreted into culture supernatants by 34 strains measured by Western blotting differed 170-fold. Sequencing the entire *agr *locus (n = 9) revealed that some had allelic variations regardless of the amount of TSST-1 produced whereas sequencing the *sar*, sigma factor B and the *tst *promoter region revealed no significant changes.

**Conclusion:**

The amounts of TSST-1 produced by clinical MRSA isolates varied. The present results suggest that TSST-1 production is not directly associated with the *agr *structure, but is instead controlled by unknown transcriptional/translational regulatory systems, or synthesized by multiple regulatory mechanisms that are interlinked in a complex manner.

## Background

*Staphylococcus aureus *(*S. aureus*) is responsible for many nosocomial and community-acquired infections. Its pathogenicity is attributed to its ability to produce many membrane-associated components and extracellular substances, several of which have been implicated as virulence factors [1,2]. One of the most unique manifestations among the various staphylococcal infections is staphylococcal toxic shock syndrome (TSS). The associated toxin TSS toxin-1 (TSST-1) is encoded by the *tst *gene, and might also be involved in the genesis of some autoimmune diseases [1,3,4]. The accessory gene regulator (*agr*) operon among several potentially associated factors is thought to positively regulate TSST-1 production [2,3]. The *agr *locus comprises 5 genes (AgrA, AgrB, AgrC, AgrD, and *hld*) that function in both transcription and translation to regulate numerous toxins, enzymes and cell surface proteins. A polymorphism in a variable region of the *agr *locus comprises nucleotide sequences encoding AgrD, the C-terminal two-thirds of AgrB, and a portion of the N-terminal half of AgrC, which has led to the assignation of *S. aureus *isolates into four classes [2,5]. In addition to the *agr *polymorphism, mutations of wild-type *S. aureus *strains resulting in *agr *deletions alter exoprotein biosynthesis [6]. However, the relationship between the *agr *polymorphism and TSST-1 production is unknown.

We previously analyzed images from two-dimensional electrophoresis (2-DE) and found that two clinical methicillin-resistant *S. aureus *(MRSA) isolates produce relatively large amounts of superantigenic exotoxins [7]. Since the amount of toxins produced is probably directly related to the virulence of *S. aureus*, evaluating the concentration of toxins produced by each strain might be useful for controlling infection.

The aim of this study was to determine whether TSST-1 production varies among clinical MRSA strains and whether it is related to variations in *agr *class and structure.

## Results

### Detection of the *tst *gene and *agr *classes

We detected the *tst *gene in 115 (75.7%) of 152 strains after PCR amplification. Among them, 53 of 66 strains from the nation-wide collection (80.3%) and 62 isolated from 86 blood samples (72.0%) harbored the gene. We identified 147 of 152 isolates (96.7%) as *agr *class 2, and 3 isolates as *agr *class 1 (1.9%). We did not identify any isolates of *agr *classes 3 or 4. The classes of 2 strains were unidentifiable. Among 112 *tst*-positive strains, 111 belonged to *agr *class 2. These results indicated the clonal dissemination of a specific group of *tst*-positive and *agr *class 2 MRSA in Japanese hospitals.

### Evaluation of TSST-1 production

We measured the amount of TSST-1 produced in 34 randomly selected strains. The densities of the bands detected by Western blotting correlated in a semi-log manner with the amount of rTSST-1 produced. The amounts of TSST-1 secreted into culture supernatants evaluated by comparison with the standard curve ranged from 0.8 to 14.0 μg/ml. Thus, the amount of TSST-1 produced varied 170-fold among clinical MRSA isolates that were cultured under the same conditions.

### Sequencing of the *agr *operon

To determine how the structure of the *agr *locus influences the amount of TSST-1 secretion, we sequenced this region in strains 1, 2, 3, 7, 8, 9, 10, 11 and 16, which generated a TSST-1 concentration range of 0.8 to 14.0 μg/ml (Table 1).

**Table 1:** Production of TSST-1 evaluated by Western blotting.

No.	Strain	μg/ml	No.	Strain	μg/ml
**1**	**N315**	3.5 ± 0.22	**18**	**2680**	1.4 ± 0.19
**2**	**A36**	14 ± 1.01	**19**	**2681**	1.3 ± 0.05
**3**	**3429**	5 ± 0.12	**20**	**2682**	1.0 ± 0.25
**4**	**3472**	1.3 ± 0.31	**21**	**2683**	1.0 ± 0.01
**5**	**3337**	1.1 ± 0.20	**22**	**2684**	0.8 ± 0.02
**6**	**1785**	1.2 ± 0.02	**23**	**2685**	1.6 ± 0.23
**7**	**2271**	2.0 ± 0.03	**24**	**2686**	2.0 ± 0.18
**8**	**3281**	4.0 ± 0.22	**25**	**2687**	1.6 ± 0.22
**9**	**2932**	2.8 ± 0.19	**26**	**2688**	7.6 ± 0.07
**10**	**3543**	14 ± 1.21	**27**	**2689**	9.8 ± 0.28
**11**	**3573**	12 ± 0.20	**28**	**2690**	3.1 ± 0.16
**12**	**V432**	7.0 ± 0.25	**29**	**2701**	5.0 ± 1.12
**13**	**V637**	7.6 ± 0.30	**30**	**2702**	2.8 ± 0.23
**14**	**V666**	5.2 ± 0.11	**31**	**2165**	4.0 ± 0.13
**15**	**V700**	8.0 ± 0.21	**32**	**3624**	1.0 ± 0.19
**16**	**V723**	1.3 ± 0.34	**33**	**3878**	2.2 ± 0.20
**17**	**V694**	4.0 ± 0.22	**34**	**3890**	6.4 ± 0.08

Strain 1, genome strain. Strains 2 to 34 were randomly selected clinical MRSA isolates that were all *tst*-positive and assigned to *agr *class 2 by PCR. Amounts of TSST-1 varied among strains and ranged from 0.8 to 14 μg/ml.

A comparison of the nucleotide sequences from the 9 strains with the corresponding sequence of the *agr *class 2 reference strain *S. aureus *SA502A (GenBank accession no., AF001782), revealed no relevant changes in the *agr*D and *agr*B regions, whereas 4 strains had allelic variations in the coding region of *agr*C, which is the receptor for two component regulatory systems. Strain 3 had a point mutation at nucleotide position 28 of the coding region that replaced phenylalanine with isoleucine. Strain 10 also had a point mutation at nucleotide position 651 of the coding region that replaced glutamine with histidine. Strain 8 had a 9-nucleotide deletion (nt 495 to 504 of the *agr*C coding sequence) that resulted in the deletion of leucine, lysine and isoleucine. Strain 2 had a nucleotide insertion that caused a frame-shift mutation, which in turn generated numerous stop codons. Although both strains 10 and 2 produced large amounts of TSST-1, the *agr *locus did not consistently vary in any way from that of the other strains (Table 2). We also sequenced the promoter regions of the *tst *gene, *sar *(staphylococcal accessory regulator) and the entire region of sigma factor B of these 9 strains. The *sar *is another positive regulatory locus for TSST-1 production that is required for maximal *agr *expression and sigma factor B is an important factor that feeds into the global regulatory network governing the expression of accessory genes [2,8-10]. No relevant nucleotide changes were evident in the sequences of both promoter regions of the *tst *gene and *sar *as well as the entire sigma factor B region (Table 2).

**Table 2:** Summary of nucleotide changes and predicted outcomes of mutations in the *agr *locus.

Strain number	Amount of TSST-1 produced (μg/ml)	Changes in *agr*C region nucleotide sequence	Predicted outcome	*tst *promoter	*sar*A	*sig*B
**1**	3.5	NC		NC	NC	NC
**2**	14	T(321) insertion	Frameshift→Truncated AgrC	NC	NC	NC
**3**	5	T 281A	phe→ile	NC	NC	NC
**7**	2	NC		NC	NC	NC
**8**	4	Δ495~504	Deletion of leu-lys-ile	NC	NC	NC
**9**	2.8	NC		NC	NC	NC
**10**	14	G651T	glu→his	NC	NC	NC
**11**	12	NC		NC	NC	NC
**16**	1.3	NC		NC	NC	NC

Data are from DNA sequencing of *agr *loci, *tst *promoter region, *sar*A and *sig*B from 9 strains. All mutations were found in *agr*C. NC, no change.

## Discussion

The proportion of *tst*-positive isolates among clinical MRSA isolates varies from < 20% to 90% according to country and clinical background [11,12]. The present study found that over 75% of clinical MRSA isolates carried the *tst *gene. This ratio is compatible with that of recent reports from Japan and it is obviously higher than those of other countries [11,12]. The ratio of *tst*-positive isolates is increasing annually and thus it is important to understand how TSST-1 production is regulated.

The mere presence of a toxin gene does not mean that the protein will be expressed and if it is, toxin levels could widely from strain to strain. In fact, the quantity of Panton-Valentine Leukocidin (PVL) produced in vitro varies up to 10-fold among MRSA strains [13].

In the present study, we identified a 170-fold difference in the amount of TSST-1 produced among MRSA isolates by Western blotting. Expression of the *tst *gene is activated by *agr *so we sequenced the *agr *locus of various TSST-1 producers to determine whether it is associated with variations in TSST-1 production. Allelic variations in the *agr*C region were identified irrespective of the amount of TSST-1 produced. One producer of a relatively large amount of TSST-1 had an insertion of nucleotides in the *agr*C that resulted in a frameshift, which in turn generated many stop codons. Other strains had allelic variations that resulted in replacement of an amino acid irrespective of the amount of TSST-1 and a frameshift in the *agr*C of a high producer was predicted to generate truncated AgrC. Therefore, the *agr *locus is probably not functional with respect to TSST-1 production in those strains. Recent findings have shown that about 25% of 105 human isolates are deficient in the production of delta-toxin, indicating that *agr *mediated regulation is disrupted [14,15]. These facts imply that mechanisms other than the *agr *locus are involved in TSST-1 production in our isolates. We also tried to evaluate *tst *gene expression by Northern blotting, but the results were not reproducible, perhaps because of high levels of expression or difficulty in removing nuclease contamination. In addition, the sequences of both the promoter region of the *tst *gene and the entire *sar *locus were conserved among these strains, indicating that these regions are not associated with variations in the amount of TSST-1 production.

The previous and present results indicate that unknown transcriptional/translational regulatory systems control TSST-1 production or that multiple regulatory mechanisms are linked in a complex manner to synthesize and produce toxin. Moreover, secretion mechanisms and proteolytic degradation would also be involved in the amount of TSST-1 produced.

A recent study has shown that variation in the amount of extracellular PVL does not correlate with the severity of infection [13]. In addition, Pragman and Schlievert noted that the transcriptional analysis of virulence regulators in animal models in vivo or in human infection do not correlate with transcriptional analysis accomplished in vitro [16]. From these viewpoints, further investigation is required to determine whether different amounts of TSST-1 are produced *in vivo *and if so, whether they are related to clinical symptoms of diseases.

## Conclusion

The present results suggest that TSST-1 production is not directly associated with the *agr *structure, but is instead controlled by unknown transcriptional/translational regulatory systems, or synthesized by multiple regulatory mechanisms that are interlinked in a complex manner.

## Methods

### Bacterial strains

Of 152 clinical MRSA isolates that we analyzed, 66 were randomly selected from the nationwide MRSA collection representing various regions of Japan in 2003, and the remainder was isolated from the bloodstream of patients in different wards at a university hospital between 1996 and 2003.

### Detection of the *tst *gene and *agr*-genotyping by PCR

Bacterial chromosomal DNA was extracted after overnight growth on Luria Bertani agar as described [17]. We detected the *tst *gene by PCR amplification using the specific primers, TGT AGA TCT ACA AAC GAT AAT ATA AAG GAT (forward) and ATT AAG CTT AAT TAA TTT CTG CTT CTA TAG TT (reverse). Genes were amplified by denaturation for 5 min at 94°C followed by 30 cycles of 30 s at 94°C, 30 s at 52°C, 60 s at 72°C and a final extension at 72°C for 5 min in a 25-μl mixture, comprising 1 μl template DNA, 0.2 mM dNTP mix, 1.5 mM 10× Ex buffer (Takara, Tokyo, Japan), 1.25 U Ex Taq (Takara) and 0.5 μM each of the forward and reverse primers. The *agr *class was determined by PCR amplification of the hypervariable domain of the *agr *locus using specific oligonucleotide primers as described [18].

### Preparation of recombinant partial TSST-1 and anti TSST-1 antibody

Fragments of the *tst *gene DNA were amplified by PCR using primers with *Bgl*II-*Hind*III restriction sites (Table 3). Amplified 280-bp DNA fragments were subcloned into the pBluescriptII plasmid, digested with *EcoR*V and transformed into *Escherichia coli *DH5α, which was then digested with *Bgl*II and *Hind*III. The *Bgl*II *-Hind*III fragment of *E. coli *DH5α was subcloned into the *BamH*I-*Hind*III site of pQE30 (Qiagen, Hilden, Germany) and transformed into *E. coli *JM109. His-tagged recombinant partial TSST-1 protein (rTSST-1) was expressed in *E. coli *JM109 and the cells were lysed using a French press (SLM Instruments, Inc., IL, USA). Recombinant TSST-1 was purified from the cell lysate using Ni-NTA agarose (Qiagen) according to the manufacturer's instructions. Purified rTSST-1 (100 μg/ml) was emulsified with an equal volume of Freund's complete adjuvant (Difco, NJ, USA) and subcutaneously injected into Japanese white rabbits to generate anti-TSST-1 antiserum. A second antibody response was elicited by immunization with the antigen alone and serum was collected.

**Table 3:** Primers used in this study.

Primer	Sequence (5'-3')	Primer	Sequence (5'-3')
2-1	AAA AAG CCA GCT ATA CAG TG	2–8	AGT GAG GAG AGT GGT GTA AAA
2-2	CTG AAT TAC TGC CAC GTT CT	2–9	TCC GTT GTT ATT TAT GCA CCT
2–3	CGA AT TCC ATA GGC TTT TC	2–10	AGA AAG GTG TGT AGC ATA TGG
2–4	GCC TTT TAT CTC ACG TCG TT	2–11	TCC TGC AAT ACT CTT ACC AT
2–5	TTC TTA CCA AATATG TCG CC	2–12	CGA GAA TCT TAA AGT ACG TGA
2–6	AAA AGT GGC CAT AGC TAA GT	2–13	CGA AGA CGA TCC AAA ACA AA
2–7	AGG TGC ATA AAT AAC ACG G	2–14	GAT TGA ATT TGA ACG TGG AG
tst forward	TGT AGA TCT ACA GAT TTT ACC CCT GTT	tst reverse	ATT AAG CTT CGC TAG TAT GTT GGC TTT

### Quantitation of TSST-1 by Western blotting

Strains were incubated in 3 ml LB broth overnight and then 100 μl cultures were transferred into 5 ml fresh LB broth and incubated at 37°C with rotary shaking at 150 rpm. Bacterial growth was monitored until the cell density reached the early stationary phase. Culture supernatant was obtained by centrifugation at 8000 × g for 15 min to precipitate bacterial cells. Total exoproteins precipitated from the culture supernatant with 10% trichloroacetic acid (TCA) were washed with cold acetone and dissolved in 100 μl of Laemmli sample buffer [19]. Proteins were resolved by electrophoresis and then Western blotted according to standard procedures with the minor modification described by Whiting et al [20]. Serially diluted rTSST-1 samples were western blotted to produce a standard curve. The individual experiments to determine TSST-1 expression for each strain were repeated three times. The density of each immunostained band was evaluated using Imagemaster 1D Elite ver.3.00 (Amersham Bioscience, Tokyo, Japan) and mean values were adopted.

### Sequence analysis of a variant *agr *locus

Table 1 lists the specific primers used to sequence the entire region of *agr *A, B, C, and D. The region was amplified by PCR under the same conditions as described for detection of the *tst *gene. The products were purified using a QIAquick PCR purification kit (Qiagen) and sequenced on a CEQ 2000 DNA analysis system (Beckman Coulter, Fullerton, CA, USA) using Beckman Dye terminator cycle sequencing kits (CEQ DTCS kit, Tokyo, Japan) according to the manufacturer's instructions.

## Authors' contributions

MN carried out the molecular genetic studies, participated in the sequence alignment, performed the immunoassays and drafted the manuscript. KY prepared the anti TSST-1 antibody. AO participated in the sequence alignment. TH participated in the design of the study. YH and MO conceived the study and participated in its design and coordination. All authors read and approved the final manuscript.
